# Does Preferred Information Format Affect Consumers’ Willingness to Pay: A Case Study of Orange Juice Produced by Biotechnology

**DOI:** 10.3390/foods12112130

**Published:** 2023-05-25

**Authors:** Yang Hu, Lisa A. House, Zhifeng Gao

**Affiliations:** 1 College of Economics and Management Department, Nanjing Agricultural University, Nanjing 210095, China; 2 Food and Resource Economics Department, University of Florida, Gainesville, FL 32611, USA; lahouse@ufl.edu (L.A.H.); zfgao@ufl.edu (Z.G.)

**Keywords:** preferred information format, willingness to pay, genetically modified organisms

## Abstract

People who received a more personally relevant message were motivated to pay closer attention to the information and actively process it, which ultimately may stimulate behavioral changes. Therefore, preferred information content has been used in many disciplines to promote effective communication. However, no study has explored the impact of preferred information formats (e.g., word, infographic, and video) concerning food production. With the increasing application of biotechnology to food production, a complex topic to communicate, and evidence that consumers were willing to pay less for bioengineered foods, efficient communication was important to impact consumer preferences. The results of this study showed that consumers mostly preferred information format is writing. Providing information in video format did improve consumers’ trust in information about food biotechnology. However, receiving information in consumers’ preferred formats did not significantly change consumers’ WTP for genetically engineered orange juice.

## 1. Introduction

Technology has provided new ways to produce food and equipped us with new methods of delivering information to consumers about such innovations. Agricultural producers, policymakers, and other food supply chain members may want to communicate with the public about new production methods to influence consumers’ acceptance, for example, of bioengineered food. As the subject of bioengineering is technically complicated, those wishing to communicate may look towards methods that are more effective in communicating the information. In general, information can be communicated to individuals in written words, pictures, or videos. Multimedia have been increasingly used in marketing and advertising to communicate with customers [1]. Individual preferences for information delivery methods based on visual and verbal cognitive styles may affect the effectiveness of information delivery and ultimately affect consumers’ choices [2]. Therefore, there is a need to investigate whether consumer preference for information delivery methods affects consumers’ willingness to pay (WTP) when a preferred or unpreferred method is used in delivering the information. It is also important to determine what factors may influence consumers’ preferred information formats. 

Cognitive psychologists conducted a review of the literature on learning styles, which refers to the concept that individuals differ in regard to what mode of instruction or study is most effective for them [3]. Psychologists refer to the concept that some people are better at processing words and others are better at processing pictures as the verbalizer–visualizer hypothesis [4]. Due to the unique information-induced cognitive load, personalization has been used to promote effective communication with customers for many years [5,6]. Personalized information generally results in a more memorable, likable, and persuasive effect than non-personalized messages [7]. When people perceive a personally relevant message, information recipients are motivated to pay closer attention to the information, process this information actively, and ultimately stimulate behavioral change [8,9,10]. Currently, there exists a large body of literature related to preferred information content, especially in the field of marketing, education, and health. 

However, to our knowledge, no study has explored preferred information delivery methods concerning food production methods. With the increasing application of biotechnology in food production and the evidence that consumers are willing to pay less for bioengineered foods, efficient communication with consumers is important to change consumers’ preference for bioengineered food and identify different biotechnologies. Especially, CRISPR (Clustered Regularly Interspaced Short Palindromic Repeats), as a newer gene editing technology, is unlike many existing genetically modified (GM) products. No genes (foreign or not) are introduced with CRISPR [11]. Considering that the process involves fewer risk factors than genetic modification (transgenic or cisgenic), scientists propose that CRISPR foods will be more acceptable than GM foods. In Florida, scientists are exploring the use of both GM and CRISPR in citrus production to combat Huanglongbing (HLB, commonly referred to as citrus greening), a disease that has dramatically impacted citrus yields and increased the cost of production in Florida. If these technologies are found to be able to control or cure the disease, the way consumers react to information will influence the success of such products in the market and, thus, the recovery of the industry. 

Previous studies have already investigated the effect of information about consumers’ WTP for bioengineered food, mainly focusing on the effect of positive and negative information and the information sources for bioengineered food [12,13,14,15,16,17,18]. In most of these studies, respondents are randomly assigned to information treatment without considering each respondent’s preferred method of information delivery. This inconsistency in the preferred information delivery method and the method used in presenting the information may affect consumers’ ability to understand the information content, which further influences consumers’ WTP for bioengineered food.

Additionally, trust in information about food-related hazards may be an important determinant of public reaction to food produced with biotechnology. Research suggests that public attitudes toward emerging technologies are mainly driven by trust in the institutions promoting and regulating these technologies [19]. Generalized trust and trust in the food system tend to offset negative perceptions associated with GM food [20]. Consumers who distrust the ability of government regulation to assure the safety of GM foods were more likely to pay a premium to purchase Non-GM foods [20]. Although there are many studies related to information source credibility, fewer explored the trust of information presented in different formats. Ye [21] found that the mean credibility for information from newspapers and magazines is higher than those from TV and radio. Interest in information also strongly influences individuals’ cognition, affective response, and persistence in learning [22,23].

To fill this gap, this paper will explore how the preference for information delivery methods (e.g., preferred, nonpreferred) affects consumers’ WTP for bioengineered orange juice (OJ) when the information is presented in different methods. We include two potential bioengineered technologies that may be applied in future OJ production, GM and CRISPR. If there is a significant difference in consumers’ WTP for GM or CRISPR OJ based on the type of information they receive relative to their preference in format, the heterogeneity across mass audiences for the information preference should be considered when communicating about these biotechnologies. Other factors regarding interest in biotechnology information and the credibility of information are also discussed in this paper. The results will provide suggestions for institutions promoting and regulating GM and CRISPR technologies about how to effectively communicate with potential consumers if the goal is to improve the acceptance of GM and CRISPR food.

## 2. The Literature Review

Some studies pointed out that people’s preference was dynamic and affected the choice context they faced. Tversky and Kahneman [24] mentioned that decision-makers are characterized by perceptions attuned to changes rather than absolute magnitudes and diminishing sensitivity to changes in response to stimuli. Due to humans’ limited working memory and limited computational capabilities, people often do not have well-defined preferences; instead, they may construct preferences on the spot by structuring or restructuring the available information, which is named constructive preferences [24,25]. 

Richardson [26] argued that information coding ability and coding preference are relatively independent dimensions along which individuals may vary. He found that judgments of presentation modality (verbal versus visual information) and memory recall were significantly related to information processing preference rather than processing ability. Childers et al. [27] pointed out that one of the factors that resulted in the differences in individual information processing is their preferred processing strategies or styles, such as verbal versus visual. Sojka and Giese [28] confirmed that individuals with a high need for cognition prefer to process verbal information. Mayer and Massa [29] decompose the visual–verbal dimension into three separable facets: cognitive ability; cognitive style; and learning preference. However, Kollöffel [30] found inconsistent results with other studies. Based on the definition in their research, cognitive abilities refer to general and specific intellectual capabilities, such as spatial ability and memory, and cognitive style refers to people’s information-processing habits, which reflect stable attitudes, dominant or preferred modes of perceiving, remembering, thinking, and problem-solving. They examined the relationships between cognitive style (such as visualizers and verbalizers), cognitive abilities (spatial and verbal abilities), and learning performance. However, they did not find a relationship between the visualizer–verbalizer cognitive styles and learning outcomes. They concluded that learning results are influenced by cognitive ability (particularly spatial visualization) and the extent to which a format allows cognitive processing rather than a match between the preferred format and the format administered. 

Studies also examine the effects of consumers’ preferred information, especially on learning processes and outcomes. Schrader et al. [31] used a one-factorial experimental design to show that students in German who learned from a personalized multimedia presentation showed higher learning outcomes. Leyzberg [32] designed a system to investigate the impact of preferred robot tutoring on learning outcomes in a long-term educational interaction and found that participants who received preferred lessons from the robot tutor outperformed participants who received non-personalized lessons. Therefore, providing consumers with preferred information is an effective tool to increase interest in the material and, in turn, activate learners to put more effort into understanding domain-related content, resulting in a higher quality of learning outcomes [33,34]. However, there are no studies investigating whether giving consumers the preferred information delivery methods could affect their ability to understand information about biotechnology and change their preference for bioengineered foods. Providing information in the preferred format may increase communication efficiency with consumers about production methods. Many scientists have been investigating the effect of delivering information related to biotechnology on consumers’ WTP for bioengineered food [17,18,35,36,37]. Hu et al. [2] randomly assigned consumers to three treatment groups, given an introduction regarding CRISPR and GM biotechnology by text, infographic, or video, and found that respondents receiving infographic and video information are willing to pay more for CRISPR orange juice (OJ) compared to GM OJ. However, they do not consider the influence of consumers’ preferences for media formats. 

Information credibility is another important factor that can affect consumers’ behavior. Previous studies investigated the effect of consumers’ trust in the bioengineered food label and information sources on consumers’ WTP. Considering trust as a prerequisite for risk communication about emerging technologies such as GM food, scientists pointed out that trust in the information affects consumers’ decisions and WTP for GM food [38]. Vecchio and Annunziata [39] pointed out that more trust in the biotechnology labels increased consumers’ WTP.

Bos et al. [40] studied the emergence of trust in a social dilemma game in four different communication situations: face-to-face; video; audio; and text chat. Participants’ trust in the first three situations was significantly improved over the text chat situation. Video and audio-conferencing groups were nearly as good as face-to-face, but both did show some evidence of what they term delayed trust (slower progress toward full cooperation) and fragile trust (vulnerability to opportunistic behavior). Although previous studies have explored the trustworthiness effect on consumers’ WTP for genetically engineered food, especially the trust in the institutions promoting or regulating these technologies, no studies provide empirical results for policymakers on which information format has the highest credibility.

## 3. Material and Methods 

**Figure 1:**
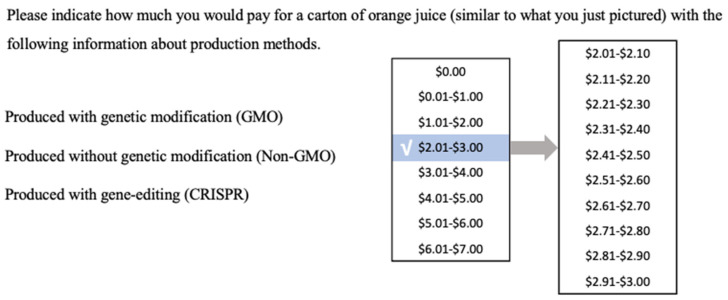
Example for payment card contingent valuation question (a second drop-down showing prices in 10-cent intervals followed the initial choice).

A survey was distributed in September 2019 through an online opt-in panel company (Toluna) to gather data with a sample of 609 respondents from primary grocery shoppers in the U.S. (including those that share the responsibility with another household member). Respondents were required to be 18 years old or older, pass a low-incidence screener [41], and correctly identify sounds from a video. Before investigating consumers’ WTP, all respondents were asked to select whether they preferred to learn information via word, infographic, or video. Respondents were randomly assigned to one of three information treatments that used different methods (word, infographic, or video) to present the same information (Appendix A.1). The Treatment Group consisted of the individuals who received the information format that was consistent with their initial preference. The respondents in the Control Group were randomly shown a different information delivery method compared to their preference. The survey flow was shown in Appendix A.2. Respondents’ WTP for OJ with three different labels (Non-GM, GM, and CRISPR) was elicited by the payment card contingent valuation method (Figure 1). The payment card contingent valuation method (CVM) is used to reveal consumers’ WTP by allowing respondents to select a point or a price interval from a list of prices [42]. Compared with other CVMs, payment card reduces missing values and avoids the boundary issue and initial bidding number issue [43,44,45]. Therefore, qualified respondents were asked to select a price interval to represent their WTP for each OJ. Based on the current retail market price of OJ in the United States, which is USD 3.67 for a carton (52–64 oz.), payment cards included USD 1.00 price intervals starting at 0 and continuing to USD 7.00. Respondents were first asked to select a price that they were willing to pay each OJ before receiving the information. After receiving the information presented in one of the formats, one question was asked to measure respondents’ understanding of the information. Then, all respondents are asked to respond to a series of questions related to GM and CRISPR to measure their knowledge of biotechnology. To measure consumers’ prior knowledge of GM and CRISPR in food production, respondents were asked to answer eighteen statements about biotechnology as true, false, or unsure [11]. A higher value represents respondents with a higher level of knowledge of GM or CRISPR. Next, all respondents were asked to state their WTP for each product in the same manner as before the information treatment. Finally, respondents answered questions about their trust and interest in the information content using a five-point Likert scale (1 = “Strongly Disagree”, 5 = “Strongly Agree”, Appendix B). Since this is the first time respondents were asked to express their trust and interest in the information, we call it the “First Stage”. Respondents were then shown the information in the two formats they had not yet received. After watching information in all three formats, respondents were asked whether the information delivery methods affected the trustworthiness of the information. They were also asked to state their trust in each specific information delivery method (Appendix B). Since this was after they received the information in all formats, we called this the “Second Stage”. At the end of the survey, sociodemographic information was collected.

### Data Analysis

To investigate the impact of information preference on consumers’ WTP for each OJ, ${WTP}_{ikt}^{*}$ for individual i for the $k^{th}$ product was asked both before and after the information treatment. t=1 represents the period before information treatment, and t=2 is the period after information treatment. A difference-in-difference analysis is used to estimate the effect of the information on the changes in WTP. Specifically, in this paper, the treatment effect on the $i^{th}$ (i=1…609) individual’s WTP for the $k^{th}(k=1\ldots 3)$ product (GM OJ, Non-GM OJ, CRISPR OJ) is estimated by the following:

(1) $${\Delta WTP=WTP}_{ik,t=2}-{WTP}_{ik,t=1}=\beta_{0}+\beta_{1}OneFormat+\beta_{2}Infographics+\beta_{3}Video+{\beta_{4}X}_{i}+\varepsilon_{ik}$$

where $X_{i}$ represents a vector of control variables for individual $i^{th}$, including measures of knowledge of GM and CRISPR, credibility, and interest in the information.

Variable OneFormat is equal to 1 if the respondents receive the information presented in a format that is the same as their initial preference; otherwise, 0. The estimated value $\beta_{1}$ represents the causal effect of consistent information format on consumers’ WTP. Variables Infographics and Video represent indicators of whether the respondents originally received information in an infographic or video format. Word format is treated as a base level.

Logistic regression is used to estimate the effect of demographics on the preferred information formats. $Y_{indic,k}=1$ represents respondents’ preferred the $k^{th}$ delivery method (word, infographic, or video); otherwise, $Y_{indic,i,k}=0$. Logistic regression is estimated by the following:

(2) $$Y_{indic,i,k}=\beta_{0}+\beta_{1}Z_{i}+\varepsilon_{ik}$$

where $Z_{i}$ represents a vector of demographic variables for individual $i^{th}$.

## 4. Results

Of the 609 qualified participants, 40% of respondents initially preferred the word information format. About 39% of respondents preferred infographics before receiving any information, and 21% of the respondents’ initial preference was video. There were 204 participants in the Treatment Group (received the information in the format of their initial preference), and 405 were in the Control Group (did not receive their preferred information format). Descriptive statistics of the sample are summarized in Table 1. To measure consumers’ prior knowledge of GM and CRISPR in food production, respondents were asked to answer eighteen statements about the biotechnology as true or false, or unsure [46]. A higher value represents respondents with a higher level of knowledge on GM or CRISPR. The mean knowledge score for GM in the Treatment Group and Control Group are 5.0 and 4.9, respectively, while the mean knowledge for CRISPR in the Treatment Group and Control Group are 2.5 and 2.4, respectively. Based on the Mann–Whitney test, there exists no significant difference between the two groups for either GM or CRISPR. The knowledge for GM is significantly higher than CRISPR in both groups based on the Wilcoxon signed-rank test. This is consistent with expectations as GM products have been commercialized for a longer time than CRISPR products; thus, consumers are expected to be more familiar with GM technology. 

After receiving the information in one format (First Stage), the mean value of consumers’ interest in the information was 3.61 in the Treatment Group and 3.72 in the Control Group. There is no significant difference in consumers’ interest between the two groups using the Mann–Whitney test. The mean value of consumers’ trust in the information in the Treatment Group and Control Group are 3.64 and 3.58, respectively. There is also no significant difference in information credibility between the groups by using the Mann–Whitney test. Therefore, receiving information in the way that consumers prefer does not affect their interests and the credibility of the information. 

After receiving the information in any one format, 61.7% of the respondents correctly answered the question measuring whether or not they understood the information contents in the Treatment Group, and 63.7% of the respondents correctly answered the question in the Control Group, indicating little difference in learning efficiency between groups. Therefore, receiving information in consumers’ preferred formats does not significantly increase their learning efficiency.

After all the respondents were given the information in all three formats (Second Stage), 69.6% and 67.4% of respondents in the Treatment and Control Groups, respectively, expressed that information delivered by different methods affected their trust in the information. Respondents’ trust in the video is significantly higher than trust in the information shown by infographics and words using the Wilcoxon signed-rank test in both the Treatment and Control Groups. 

Comparing information credibility in the Treatment Group receiving the information in one format (First stage) with receiving the information in all formats (Second stage), the median value significantly decreases from 4 (Trustworthy) to 2 (A little trustworthy) with a *p*-value 0.00 by using Wilcoxon Test. In the Control Group, the median information credibility after receiving the information in one format was 3 (Neutral), while the credibility after receiving the information in all formats became 2 (A little trustworthy). There is a significant difference between the median trust value in the first stage and the median trust value in the second stage, with a *p*-value of 0.00 via using the Wilcoxon Test. Therefore, no matter whether respondents received information in their preferred way or not, presenting multiple media simultaneously lowers consumers’ trust in the information content. 

The effect of information format preference on consumers’ WTP estimations is shown in Table 2. Respondents who receive information in the same format as their original preferred format (OneFormat = 1) change their WTP for Non-GM OJ USD 0.19 more than respondents that do not receive their preferred format (OneFormat = 0). For GM OJ, consumers who trust the information content more will be willing to pay USD 0.17 more than consumers who have lower trust in the information content. Respondents with higher knowledge of GM technology are willing to pay USD 0.06 more than consumers who have lower knowledge. Respondents who trust the information content more indicate a USD 0.11 higher WTP for CRISPR OJ. Respondents who receive information by infographics will be willing to pay USD 0.33 more for CRISPR OJ than consumers who receive information by word.

To investigate factors that may affect consumers’ preferred information formats, the results of logistic regressions are shown in Table 3. For the preference of word information, the marginal effect of gender is −0.12, indicating that males are 12% less likely to prefer the word format compared to females. Respondents who are 45–54 years old are 12% more likely to prefer the word information format compared to respondents who are 18–24 years old. Compared to respondents who have full-time jobs, students and retired respondents have a 28% lower and 20% higher probability of preferring the word information format, respectively. Respondents who like gardening are 3% more likely to prefer the word information format. Respondents following the celebrities have a 5% lower probability of preferring the word information format. Respondents who pay more attention to the government and politics are 5% more likely to prefer the word information format. Compared to respondents living in urban areas, respondents living in suburban areas are 9% less likely to prefer the word format. 

Respondents who are 45–54 years old are 15% less likely to prefer the infographic information format compared to respondents who are 18–24 years old. Retired respondents are 19% less likely to prefer infographic information than respondents who have full-time jobs. Respondents who like gardening are 3% less likely to prefer infographic information than respondents who do not like gardening. Respondents following the celebrities are 5% more likely to prefer the infographic information format. Respondents who pay more attention to the government and politics have a 4% lower probability of preferring the infographic information format. Compared to respondents living in urban areas, respondents living in suburban and rural areas are 11% and 10% more likely to prefer the infographic format, respectively. Respondents whose income is from USD 25,000–34,999 and USD 75,000–99,999 have an 18% higher probability of preferring infographic information than respondents whose income is less than USD 14,999. 

For the preference of video information format, gender, having children in the household, and income significantly affect the probability of preferring the video format. Males are 11% more likely to prefer the video format compared to females. Households without children are 10% less likely to prefer the video information format than households with children. Respondents with incomes ranging from USD 25,000–34,999 and USD 100,000–149,999 have a lower probability of preferring video information than respondents whose income is less than USD 14,999.

## 5. Discussion

Changes in WTP for GM and CRISPR OJ did not differ for consumers who received their preferred information format compared to those that did not. Thus, the results suggest that there is no need to consider consumers’ preference for information format when institutions communicate about biotechnology with consumers to improve their WTP for GM or CRISPR food. The result is consistent with some prior studies. Massa and Mayer [4] pointed out that there was no strong support for the hypothesis that verbal and visual learners should be given different kinds of multimedia instruction. Learning styles might be a neuromyth, a misconception about brain neurofunction patterns [3,47]. The individually preferred way of learning is often a bad predictor of the way people learn most effectively. Until now, there has been no scientific evidence that learners with different learning styles should be taught with different instructional methods [47]. Thus, even though respondents received the information about biotechnology in their preferred format, it did not improve their learning efficiency, which further did not change their preference for GM or CRISPR OJ. 

For the preference of information format, our results show that consumers prefer information presented in word and infographic formats over the video format. Gender, having children in the household, age, occupation, gardening, following celebrities, attention to government and parties, types of community, and income are the factors significantly affecting the consumers’ preferred information format. The results show that retired respondents, people who garden, and those that pay more attention to government and politics are more likely to prefer the word format and less likely to prefer infographics. Respondents following celebrities and living in suburban areas have a higher probability of preferring the infographic format. However, none of these factors have a significant effect on the preference for the video information format. 

In this survey, more than half of the individuals express that information delivery formats have an influence on information credibility. Consumers’ trust in biotechnology information could significantly increase WTP for GM and CRISPR OJ by USD 0.17 and USD 0.11, respectively. This is consistent with the results from Vecchio and Annunziata [39] that more trust in the biotechnology labels increased consumers’ WTP. After receiving all the information (Second Stage), the video version was the most trustworthy format compared to infographic and word, though it was initially the least preferred method of delivery. Presenting information via video could improve trustworthiness, indirectly increasing consumers’ WTP for bioengineered OJ. The result is consistent with previous studies that showed that video and audio communication could significantly improve trust [40].

## 6. Conclusions

The idea of matching the individuals’ preferences and the representational format of the information materials has its appeal, especially in the eyes of psychologists and educators. Psychologists have examined the verbalizer–visualizer hypothesis that some people are verbal learners and others are visual learners. However, there is no empirical application of this hypothesis on consumers’ preference for food. This paper answers the question of whether matching information delivery formats with consumers’ preferences impacts consumers’ WTP for OJ and can inform researchers whether the preferred information format should be a larger part of experimental design for food consumption studies. 

Our findings have important implications for better communicating about agricultural biotechnology with consumers. Online media has become an important channel for obtaining information. Our results show that giving consumers the preferred information delivery methods does not affect their learning efficiency about biotechnology. As preferred information formats do not significantly change consumers’ WTP for GM and CRISPR OJ specifically, there seems to be no evidence for policymakers and food institutions to spend time, effort, and money on providing biotechnology information based on the individual preferred format. However, providing information in video format could improve consumers’ trust in the information. Therefore, policymakers and food institutions could use more video information to improve consumers’ trust in the information content, which may further improve consumers’ WTP for biotechnology foods. The limitation of this paper is that consumers’ preference is self-expression. Future studies may consider using neuroscience methods to capture emotional changes and identify changes in consumers‘ preferences.

## Figures and Tables

**Table 1:** Descriptive statistics of individuals’ characteristics.

Variable	Treatment Group	Control Group
Male	43.1%	40%
College degree and above	78.4%	76.8%
Annual Household Income		
Income < 14,999	10.8%	9.1%
Income USD 15,000–24,999	10.3%	12.6%
Income USD 25,000–34,999	11.8%	13.1%
Income USD 35,000–49,999	13.2%	18%
Income USD 50,000–74,999	21.1%	17.8%
Income USD 75,000–99,999	13.2%	13.8%
Income USD 100,000–149,999	12.8%	11.1%
Income USD 150,000–199,999	5.4%	2.5%
Income USD 200,000+	1.5%	2.0%
Knowledge of GM	5.0	4.9
Knowledge of CRISPR	2.5	2.4
Percentage of respondents correctly answer	61.7%	63.7%
After receiving information in one format (First Stage)		
Interest in the information	3.61	3.72
Trust in the information	3.64	3.58
After receiving information in all formats (Second Stage)		
Information format affect credibility	69.6%	67.4%
Trust in the information by word	3.57	3.62
Trust in the information by infographic	3.68	3.66
Trust in the information by video	3.79	3.75
Respondents’ preference for each information format		
Word	40%	
Infographic	39%	
Video	21%	

**Table 2:** Results for the estimations of the impact of preferred information format.

Variable	Model 1 Changing WTP for Non-GM OJ	Model 2 Changing WTP for GM OJ	Model 3 Changing WTP for CRISPR OJ
Pre_WTP_NonGM	NA	0.09 **	0.10 **
Pre_WTP_GM	0.09 **	NA	−0.24 ****
Pre_WTP_CRISPR	−0.11 ***	−0.17 ****	NA
OneFormat	0.19 **	−0.15	−0.02
Infographics	0.17 *	0.02	0.33 **
Video	−0.00	−0.08	0.14
GM Knowledge	0.03	0.06 **	0.05
CRISPR Knowledge	−0.02	−0.01	−0.03
Trust	−0.06	0.17 ***	0.11 *
Interest	0.01	−0.05	0.09

Note: ****, ***, **, * represent statistical significance at the 0.1%, 1%, 5%, and 10% levels, respectively.

**Table 3:** Results of logistic regression for different preferred information formats.

Variable	Word		Infographics		Video	
	Coefficient	Marginal Effect	Coefficient	Marginal Effect	Coefficient	Marginal Effect
Gender	−0.60 ***	−0.12 ***	0.07	0.01	0.71 ***	0.11 ***
No kids	0.29	0.06	0.19	0.04	−0.63 ***	−0.10 ***
Age range 25–34	−0.12	−0.03	−0.22	−0.05	0.46	0.07
Age range 35–44	0.32	0.07	−0.26	−0.06	0.01	0.00
Age range 45–54	0.60 *	0.12 *	−0.69 **	−0.15 **	0.19	0.03
Age range 55–64	0.06	0.01	−0.54	−0.12	0.74	0.12
Age range 65+	0.33	0.07	−0.26	−0.06	−0.08	−0.01
Student	−1.36 *	−0.28 *	0.49	0.11	0.47	0.07
Retired	0.98 ***	0.20 ***	−0.86 **	−0.19 **	−0.36	−0.06
Other employment	0.10	0.02	−0.04	−0.01	−0.04	−0.01
Gardening	0.12 *	0.03 *	−0.16 **	−0.03 **	0.04	0.01
Following celebrities	−0.23 **	−0.05 **	0.22 **	0.05 **	−0.01	−0.00
Frequency party	0.23 ***	0.05 ***	−0.18 **	−0.04 **	−0.06	−0.01
Living in suburban areas	−0.42 **	−0.09 **	0.52 **	0.11 **	−0.16	−0.03
Living in rural areas	−0.20	−0.04	0.48 *	0.10 *	−0.42	−0.07
Income USD 15,000–24,999	−0.30	−0.06	0.43	0.09	−0.19	−0.03
Income USD 25,000–34,999	−0.05	0.01	0.83 **	0.18 **	−1.18 **	−0.18 **
Income USD 35,000–49,999	−0.18	−0.04	0.58	0.13	−0.53	−0.08
Income USD 50,000–74,999	−0.12	−0.03	0.53	0.11	−0.50	−0.08
Income USD 75,000–99,999	−0.57	−0.12	0.88 **	0.18 **	−0.42	−0.07
Income USD 100,000–149,999	0.15	0.03	0.55	0.12	−0.99 **	−0.16 **
Income USD 150,000–199,999	−0.50	−0.10	0.17	0.04	0.30	0.05
Income USD 200,000+	0.01	0.00	0.64	0.14	−0.86	−0.13

Note: ***, **, * represent statistical significance at the 1%, 5%, and 10% levels, respectively.

**Figure A1:**
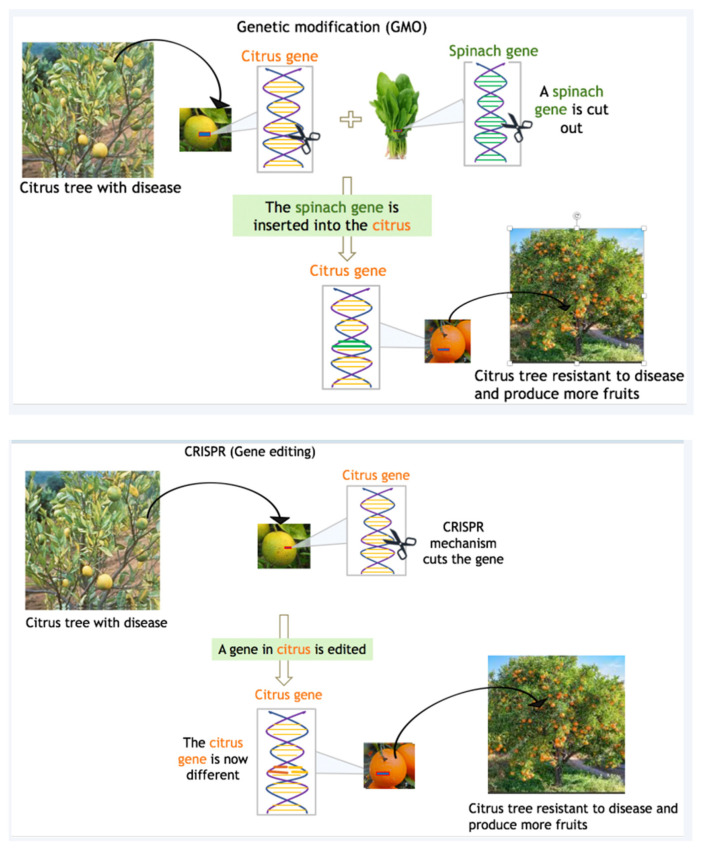
II. Infographic Group.

**Figure A2:**
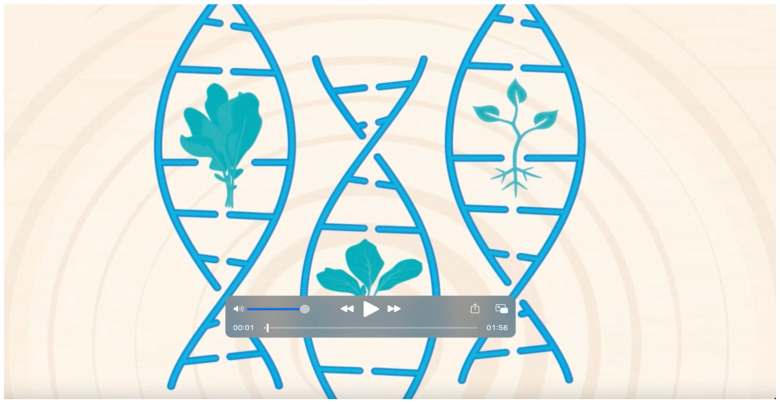
III. Video Group.

**Figure A3:**
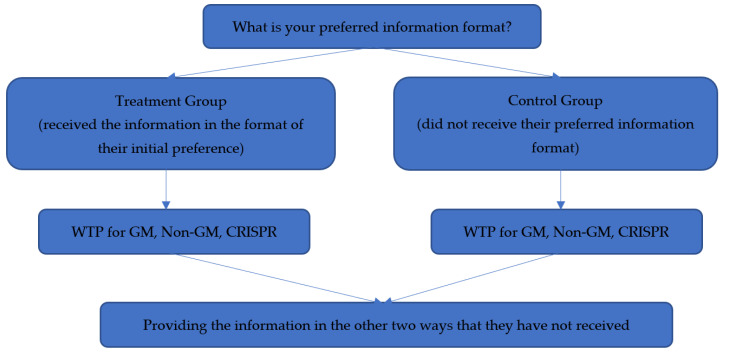
Survey flow.

## Data Availability

The data presented in this study are available on request from the corresponding author.

## Appendix A

### Appendix A.1. Information Treatment

The following paragraphs explain in more detail the difference between genetic modification and CRISPR (gene-editing):

I. Word Group

1. Genetic modification, or GM, is a way to improve crop varieties. GM involves adding a gene from one species into a different species, giving it new or different characteristics. The new gene becomes part of the GM plant. For example, a gene from spinach could be inserted into a fruit plant to help the fruit plant resist a disease and produce more fruit.
2. Gene-editing, such as CRISPR, is a way to improve crop varieties. CRISPR works like a word-processor to edit the text of the DNA instruction manual to improve the directions the plant receives. CRISPR can accurately target a specific part within a gene to be altered, causing a break in the DNA and using the cell’s natural repair machinery to introduce changes, similar to a search and replace when editing text. For example, the genes of a fruit could be edited to produce a fruit plant that can resist a disease and produce more fruit.

## Appendix B

Please indicate how much you agree with the following statements:

**Table A1:** Consumers’ interest and trust in the information content.

	Strongly Disagree	Disagree	Neither Agree nor Disagree	Agree	Strongly Agree
I am interested in the information about biotechnology that I saw earlier in the survey	○	○	○	○	○
I trust the information about biotechnology that I saw earlier in this survey	○	○	○	○	○

**Table A2:** Trustworthiness of the information delivery methods.

Do you think the method of delivering information (such as a written description, images, and videos) affects the trustworthiness of the information about biotechnology?					
○ Yes (1) ○ No (2)					
How trustworthy did you find the information in each description?					
	**Not at all Trustworthy**	**A little Trustworthy**	**Neutral**	**Trustworthy**	**Very Trustworthy**
Written description	○	○	○	○	○
Images/Visual description	○	○	○	○	○
Video	○	○	○	○	○
